# Clinical Application of the HCM-AF Risk Score in the Prediction of Clinical Outcomes of Polish Patients with Hypertrophic Cardiomyopathy

**DOI:** 10.3390/jcm12134484

**Published:** 2023-07-04

**Authors:** Maria Stec, Agata Suleja, Daniel Gondko, Wiktoria Kuczmik, Jakub Roman, Dominika Dziadosz, Krzysztof Szydło, Katarzyna Mizia-Stec

**Affiliations:** 1 Students’ Research Group of the 1st Department of Cardiology, Medical University of Silesia, 47 Ziołowa St., 40-635 Katowice, Poland; mariaannastec@gmail.com (M.S.); agatasuleja@gmail.com (A.S.); gondko.daniel@gmail.com (D.G.); wikikuczmik@interia.pl (W.K.); grrom98@gmail.com (J.R.); 2 1st Department of Cardiology, Medical University of Silesia, European Reference Network of Heart Diseases (ERN GUARD-HEART), 47 Ziołowa St., 40-635 Katowice, Poland; dominika.dziadosz@gmail.com (D.D.); kszydlo1964@gmail.com (K.S.)

**Keywords:** hypertrophic cardiomyopathy, atrial fibrillation, HCM-AF Risk Score

## Abstract

The recently introduced HCM-AF Risk Calculator allows the prognosis of atrial fibrillation (AF) occurrence in hypertrophic cardiomyopathy (HCM) patients. The aim of this study was to assess the clinical application of the HCM-AF Risk Score in the prediction of the clinical outcomes of Polish patients. The study included 92 patients (50.0% female, median age 55 years), with a baseline sinus rhythm diagnosed between 2013 and 2018. The analysis involved the incidence of clinical characteristics and outcomes, total mortality, rehospitalisation, and the course of heart failure (HF). According to the HCM-AF Risk Score, the HCM population was stratified into three subgroups, with a low (13/14.2%), intermediate (30/32.6%), and high risk of AF (49/53.2%). Subgroups differed significantly: the high-risk subgroup was older, had a higher body mass index (BMI), and more advanced signs of left ventricular (LV) hypertrophy and left atrium (LA) dilatation. The registered AF incidence was 31.5% and 43.5% in the 2- and 5-year follow-ups, and it was significantly higher than in the HCM-AF Risk Score population, which had 4.6% in the 2-year follow-up, and 10.7% in the 5-year follow-up. In the whole population, the AF incidence in both the 2- and 5-year follow-ups revealed a strong correlation with the HCM-AF Risk Score (r = 0.442, *p* < 0.001; r = 0.346, *p* < 0.001, respectively). The clinical outcomes differed among the subgroups: the total mortality was 15.4% vs. 20.0% vs. 42.9% (*p* < 0.05); rehospitalisation was 23.1% vs. 53.3% vs. 71.4% (*p* < 0.05). The highest HF progression was in the high-risk subgroup (36.7%). Regardless of the high results of the HCM-Risk Score in Polish patients, the score underestimates the real-life high level of AF incidence. The HCM-AF Risk Score seems to be useful in the prediction of the general clinical outcomes in HCM patients.

## 1. Introduction

Hypertrophic cardiomyopathy (HCM) is a primary myocardial genetic disorder characterised by myocardial hypertrophy. Hypertrophy can be described as the structural, functional, and electrical remodelling of the myocardium. The global prevalence of HCM oscillates around 1:500 (0.2%), thus affecting approximately 894,000 patients in Europe, and 75,500 in Poland [1,2]. 

HCM is a complex and heterogeneous disease with a wide spectrum of clinical manifestations, ranging from asymptomatic individuals, to those experiencing debilitating symptoms, such as heart failure (HF), sudden cardiac death, and arrhythmias [3]. Among the arrhythmias associated with HCM, AF is the most prevalent and frequently encountered rhythm disturbance, affecting up to 18% of patients [4,5,6]. 

AF in HCM is associated with an increased risk of adverse cardiovascular outcomes. The presence of AF is an independent predictor of morbidity and mortality in HCM patients [6]. According to Konstantinos et al., the hazard ratio for the effect of AF on overall mortality in HCM was 1.48 [6]. Moreover, AF is associated with a higher risk of heart failure, stroke, thromboembolic events, and sudden cardiac death [7]. Furthermore, the burden of AF (i.e., persistent, paroxysmal, or permanent) appears to be correlated with adverse outcomes, with persistent AF carrying the highest risk [5]. 

AF constitutes an important step in the progression of HCM, with substantial clinical implications. Thus, the prediction of AF, as well as its detection and monitoring, are crucial for a comprehensive approach to patient care, improved therapy outcomes, and an enhanced quality of patient life. In recent years, Carrick et al. [8] developed and published a tool called the HCM-AF Risk Score, which allows the accurate prognosis of AF occurrence in HCM patients for the nearest 2 and 5 years. This tool takes into account four readily available and clinically relevant variables: the diameter of the left atrium (LA), the presence of HF symptoms, age at HCM diagnosis, and age at current clinical evaluation. The score enables the stratification of patients with HCM into three distinct risk groups, based on their likelihood of developing AF. These groups are classified as having low risk, intermediate risk, and high risk of AF. This score can reliably and individually stratify patients with HCM according to their risk of newly diagnosed AF, and offers the opportunity for more personalised and tailored management strategies to be implemented, potentially leading to improved patient outcomes in the long term. The HCM-AF Risk Score was developed using the Tufts Institute population of HCM patients, with a HCM diagnosis based on echocardiographic or cardiac magnetic resonance (CMR) imaging. The external validation of the tool was performed on a cohort of patients hospitalised at the Hypertrophic Cardiomyopathy Clinic at Toronto General Hospital. The tool has been developed and validated on the American population, and has proven reliable in identifying patients at risk of developing AF, with a concordance of 0.70 within the development cohort, and a concordance of 0.68 within the validation cohort, respectively [8]. 

The course of HCM significantly differs between different racial and ethnic groups [9,10]. As the Polish population differs from the American population taken into account in the mentioned study, we wanted to test the score again, but in our population [2]. 

The aim of our study was to assess the clinical application of the HCM-AF Risk Score in the prediction of the 2- and 5-year clinical outcomes of Polish patients with HCM. 

## 2. Materials and Methods

This was a retrospective cohort one-centre study. It was held at the high-volume tertiary cardiology centre First Clinic of Cardiology, at the Medical University of Silesia in Katowice, a member of the European Reference Network of Heart Diseases (ERN GUARD-HEART). 

**Figure 1:**
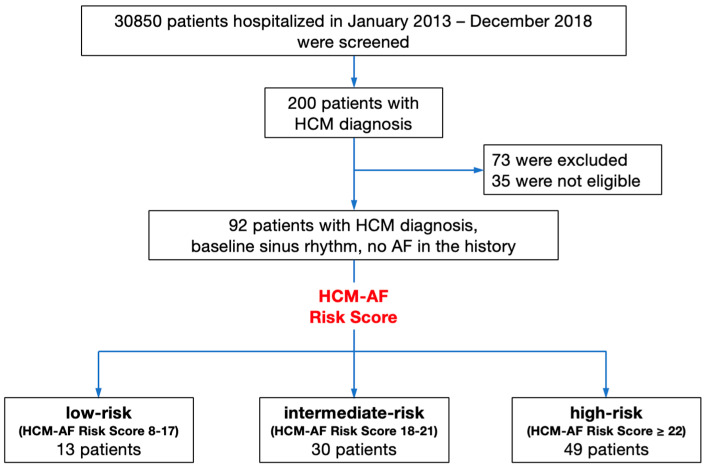
Chart of the study methodology. AF—aftrial fibrillation; HCM—hypertrophic cardiomyopathy.

All consecutive admissions to the centre between 1 January 2013 and 31 December 2018 (N = 30,850) were screened, to identify patients who were hospitalised due to a first diagnosis of HCM made at the clinic, or confirmed by echocardiography performed at the clinic. Baseline sinus rhythms were identified by scrutinising the centralised electronic medical database. Exclusion criteria were a history of AF, and a diagnosis of HCM phenocopy, such as sarcomeric HCM, Fabry disease, Pompe disease, and Danon’s disease (Figure 1.)

The analysis involved the following clinical data: sex, age at diagnosis, age at clinical evaluation, symptoms of heart failure assessed using the New York Heart Association (NYHA) Functional Classification, body mass index (BMI), implantable cardioverter-defibrillator (ICD), baseline parameters obtained using transthoracic echocardiography (TTE), and the presence of non-sustained ventricular tachycardia (nsVT) and supraventricular tachycardia (sVT) detected using electrocardiogram (ECG) or Holter monitoring. The study presented only baseline TTE parameters, including the left ventricular end-diastolic diameter (LVEDD), the diastolic interventricular septum (IVSd), posterior wall thickness (PWd), the left ventricular ejection fraction (LVEF), the global longitudinal strain (GLS), and the left ventricular mass index (LVMI). The risk of AF was calculated for each patient using the HCM-AF Risk Score.

According to the HCM-AF Risk Score, the three risk-stratification subgroups were distinguished, with low (8–17 points), intermediate (18–21 points), and high (≥22 points) risk for AF. 

The study included a 2- and 5-year clinical follow-up for all subjects, and clinical observation up to 10 years for patients who had the diagnosis before 2018. Data on the following clinical outcomes were analysed: AF occurrence (AF confirmed on 12-lead ECG or Holter monitoring), total mortality (data from the National Health System, and direct contact with immediate relatives), rehospitalisations, ICD implantation, and the stage of heart failure (HF) in the NYHA class.

The following definitions/terms important to this analysis were used:

- AF was defined as any form of AF (paroxysmal, persistent, permanent).
- Clinical endpoints of the study:
  - The primary endpoint was the AF occurrence in the 2- and 5-year follow-ups.
  - The secondary endpoints were the total mortality, rehospitalisation due to HF, and the complex endpoints including both total mortality and rehospitalisation due to HF.
- Other data on the clinical outcomes of HF include the progression of HF defined as increase ≥1 of stage in the NYHA scale; the regression of HF defined as decrease ≥1 of stage in the NYHA scale.

The study endpoints were analysed with regard to the baseline HCM-AF Risk Score.

The study was approved by the Bioethical Committee of the Medical University of Silesia. The statistical analysis was performed using the Jamovi software (Jamovi version 2.3). The data are presented as the mean ± SD, or as the numbers of patients and percentages, where appropriate. The chi-square test was used to test the relationship between the nominal variables, and check whether the compared groups were equal. The Kruskal–Wallis test was used to assess the statistical significance of differences between more than two groups. If there were such differences, an appropriate post hoc test was used. The Kaplan–Meier method was used to estimate the cumulative probability for the occurrence of an outcome. A *p*-value < 0.05 was considered statistically significant.

## 3. Results

### 3.1. Participants’ Clinical Characteristics

A total of 92 patients with the first diagnosis of HCM, baseline sinus rhythm, and no history of AF were included in the analysis. In the total cohort, the median age was 55 years, 50.0% of patients were male, and the mean BMI was 26.1 kg/m^2^. The TTE results were representative of HCM characteristics, with features of LV hypertrophy (mean LVMI: 160.0 g/m2) with preserved LVEF (mean: 52.3%) (Table 1). 

According to the HCM-Risk Score, the whole HCM population was stratified into three subgroups: a subgroup with a low risk of AF (N = 13/14.2%), a subgroup with an intermediate risk of AF (N = 30/32.6%), and a subgroup with a high risk of AF (N = 49/53.2%). 

There were some differences in the clinical characteristics of the study subgroups. Patients from the high-risk subgroup were significantly older (age at HCM diagnosis 51.8 ± 15.4, *p* < 0.001), more overweight (BMI 29.1 ± 4.6 kg/m^2^, *p* = 0.002), and presented advanced symptoms of HF more often than the low and intermediate risk subgroups. In transthoracic echocardiography at baseline, patients from the high-risk subgroup had a higher end-diastolic volume (LVEDD 49.0 ± 7.5, *p* = 0.043), and a more dilated left atrium (LA diameter 49.9 ± 9.6 mm, *p* < 0.001; LA area 32.3 ± 8.1 cm^2^, *p* < 0.001). However, a higher rate of patients from the low-risk subgroup suffered from nsVT, or had an ICD implanted in the follow-up. There was no registered sVT on the baseline ECG and Holter monitoring in the study group.

### 3.2. Registered AF Incidence and HCM-AF Risk Score

The registered AF incidence for the whole HCM population was 31.5% in the 2-year follow-up, and 43.5% at the 5-year follow-up, and it was higher compared to the AF incidence registered by the HCM-F Risk Score (4.6% in the 2-year follow-up, and 10.7% in the 5-year follow-up).

The comparison of the registered AF incidence with the estimated risk of AF revealed discrepancies in almost all subgroups; the registered AF incidence was higher than the estimated AF risk. 

In the low-risk subgroup, the AF in the 2-year follow-up was registered in 7.7% patients vs. 1.1% the estimated AF risk; in the 5-year follow-up, it was 15.4% vs. 2.6%. In the intermediate-risk subgroup, AF in the 2-year follow-up was registered in 16.7% patients vs. 3.5%, and in the 5-year follow-up, it was 40.0% vs. 8.3%. In the high-risk subgroup, AF in the 2-year follow-up was registered in 46.9% patients vs. 13.6%, and in the 5-year follow-up, it was 51.0% vs. 29.0%. (Table 2.)

### 3.3. Outcome Data

Data about the AF incidence in the 2- and 5-year follow-ups are presented above. There were differences in the registered AF incidence among the analysed subgroups with the lowest rate in the low risk subgroup, and the highest rate in the high-risk subgroup (Table 3).

The secondary endpoints in the whole HCM population were registered as follows: the total mortality was 42.6%, the rehospitalisation due to HF was 58.7%, and the complex endpoints were 64.1%.

The comparison of the secondary endpoints among the subgroups revealed significant differences in the total mortality, rehospitalisation due to HF, and the complex endpoints. The frequency of the endpoints was as follows in the high-risk subgroup: 42.9%, 71.4%, 77.6%.

The HF assessment showed progression of the symptoms in 27.2% of HCM patients. The highest rate of HF progression was in the high-risk subgroup (36.7%), and no HF progression was observed in the low-risk patients (100%) (Table 3).

**Figure 2:**
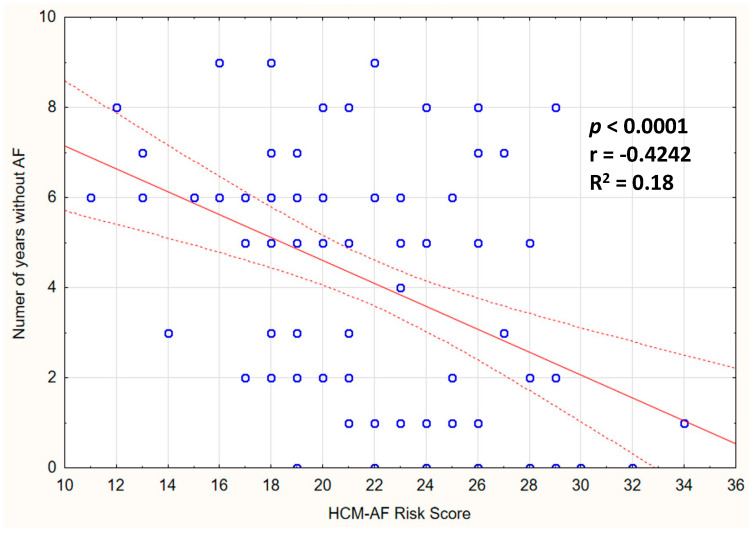
Correlation of the HCM-AF Risk Score with the number of years free from AF in HCM patients.

In whole HCM population, the AF incidence in both the 2- and 5-year follow-ups revealed significant correlation with the HCM-AF Risk Score (r = 0.442, *p* < 0.001; r = 0.346, *p* < 0.001, respectively). The correlation between the number of years free from AF, and the HCM-AF Risk Score, is presented in Figure 2.

Similarly, in the high-risk subgroup, the AF incidence in the 2-year and 5-year follow-ups corresponded with the HCM-AF Risk Score (*p* = 0.012 and *p* = 0.014, respectively). There were no significant correlations in the low- and intermediate-risk subgroups.

**Figure 3:**
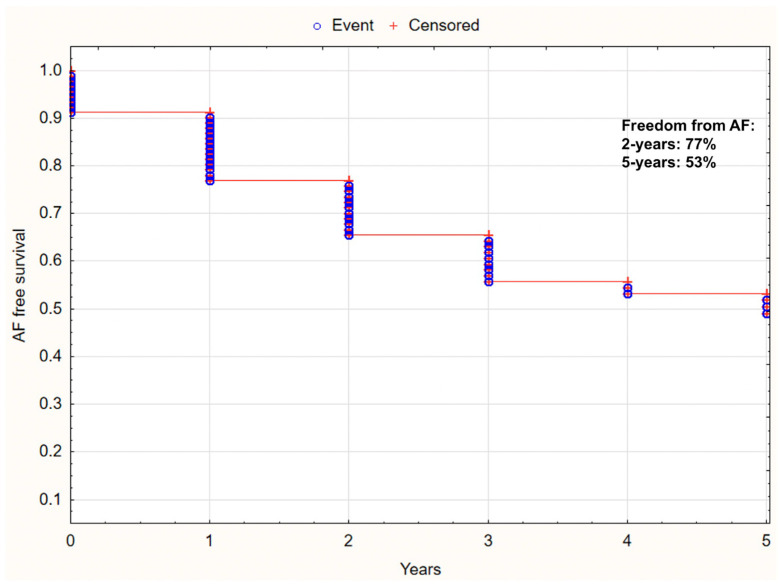
Kaplan–Meier curve for survival free from AF, in HCM patients.

The rate of survival free from AF in all the analysed HCM patients is presented in Figure 3.

## 4. Discussion

We present data for the HCM-AF Risk Score evaluation in Polish patients with HCM. The presented paper is the first evaluation of the HCM-AF Risk Score’s efficacy in a European and Polish population. Based on the retrospective analysis, we verified the AF incidence and clinical outcomes in patients with HCM, and compared the AF data with the estimated AF risk. 

The concept of our study was based on the Risk Assessment Scale by Carrick et al. [8]. In 2021, AHA Journals published a paper proposing a new classification of the risk of developing AF in patients with HCM, within 2 and 5 years [8]. 

According to the HCM-AF Risk Score scale, we both estimated the AF risk, and distinguished the three risk subgroups, with low, intermediate, and high risk. It is worth noting that the risk subgroups differed in size. However, statistical analysis was carried out thoroughly, to allow a comprehensive presentation of the results. The discrepancy in the number of participants in each risk subgroup does represent the structure of risk of AF in the Polish HCM population, and gives insight into the overall characteristics of this group. 

The prevalence of AF was high in Polish patients with HCM, with the highest prevalence in the high-risk subgroup. Regardless of the fact that Polish patients with HCM were characterised by a relatively high HCM-Risk Score, the score underestimated the real-life high level of AF incidence. 

It is worth noting that the risk subgroups differed with regard to clinical presentation and outcomes. The worst clinical outcomes, including death from any cause and/or rehospitalisation due to HF, was observed in the high-risk subgroup. Thus, our results confirm that the HCM-AF Risk Score may be a useful tool in the prediction of general clinical outcomes in HCM patients. It allows the distinction of HCM patients with high mortality and rehospitalisation due to HF. 

The age at HCM diagnosis of our population was comparable with data presented by Carrick et al. in their study, validating the HCM-AF Risk Score (46.5 ± 15.9 vs. 45 ± 17) [8]. Moreover, the study group was similar to the cross-sectional Polish population [2]. The prevalence of AF in our population was higher than in the mentioned study; in the 5-year follow-up, that study predicted that AF would affect 10.7% of the general population, whereas according to our data, the AF was present in 49% of our population (17.2% vs. 56.1% in the high-risk subgroup). This ratio also exceeds the general predictions of AF prevalence in the HCM population, which is estimated from 12.5% to 24% [11,12]. A greater percentage of the patients were qualified into the high-risk group, compared to the Carrick et al. study (53.3% vs. 35.78%). 

Specialists have estimated the risk of developing AF using this score, according to the 2014 European Society of Cardiology guidelines, predicting that a left atrial (LA) transverse dimension ≥45 mm is a strong predictor of developing this arrhythmia in the HF patient population [13]. In our study, an LA transverse dimension of more than 45 mm was particularly observed in the high-risk AF development subgroup; the mean value for this group was 49.9 ± 9.6 mm. However, the analysis clearly showed that assessing this parameter is not a strong enough predictor of AF development, because in the subgroup of medium risk, where the mean LA transverse length was 39.7 ± 4.3 mm, in the 5-year follow-up, AF developed in as many as 40% of the patients studied. 

In 2013, Alonso A. et al. presented a multicentre study validated on a U.S. and European population of patients at high cardiovascular risk, a study with a model developed to assess the risk of developing AF with the CHARGE-AF Score. The model evaluated parameters such as congestive HF, hypertension, age, diabetes, history of stroke or transient ischaemic attack, vascular disease, and female gender. The CHARGE-AF Score helps estimate the risk of AF at a specific time, with a C-statistic of 0.765, and a CI of 0.748 to 0.781 [14]. It should be noted that although the CHARGE-AF scale is widely used to assess the risk of AF in the general population, it may not be specifically validated or adapted to patients with HCM [14,15,16]. Alonso et. al. found that the European population has a significantly higher burden of developing atrial fibrillation, compared to other populations studied. Additionally, they are characterised by higher BMI values (28.7 kg/m^2^), older age (72 years), and features of left ventricular hypertrophy [14]. Our study on a population of Polish patients with HCM showed similar findings, wherein patients in the subgroup at high risk of developing AF were characterised by a higher age (63 years), BMI (29.1 kg/m^2^), and left ventricular late diastolic thickness, suggesting that Polish patients fit the characteristics of the European population. 

Yan-Guang Li et al. proposed the C2HEST AF risk assessment model, which they validated on Chinese and Korean populations in 2019. The model assessed five basic parameters: congestive heart failure, chronic kidney disease, hypertension, age, stroke or transient ischaemic attack, and thyroid disease, in patients with and without cardiovascular risk. The scale proved significantly useful in assessing risk among patients without structural heart disease, including HCM, with an AUC of 0.75, and a calibration (*p* = 0.774) [17]. In the high-risk subgroup of the C2HEST AF risk model were 0.4% of the patients studied, while in the case of our validated scale, it was 63% of patients, and AF was diagnosed in 5.3% and 51%, respectively, during the follow-up period, which may suggest that our validated scale is more sensitive in HCM patients. 

As can be seen, despite previous attempts to develop an ideal tool for assessing the risk of AF, researchers have not focused on a group at particular risk of developing this arrhythmia, which can definitely be said of patients with HCM [18], while proposing models for broader screening. 

For this reason, the problem of AF cannot be ignored. The concordance of the novel HCM-AF Risk Score was 0.7 with the external validation cohort of 0.68 [8]. Despite the promising performance of the score in the population evaluated by Carrick et al., it is key that we individualise the approach for other populations. Further studies must be conducted to include other risk factors, which may play a crucial role. The other future-proof solution that would exclude human error would be to use machine-learning methods. The HCM-AF Risk Model, which was trained by Bhattacharya et al., has 0.74 sensitivity, 0.70 specificity, and an AUC (c-index) of 0.8. They distinguished 18 variables that are correlated with the occurrence of AF in HCM patients. According to Bhattacharya et al., the diameter of the LA was the strongest independent predictor of AF, alongside age and NYHA class. Moreover, AF was also identified to correlate positively with, e.g., undergone septal myectomy, applied diuretic treatment, and dyspnoea upon exertion; and negatively with, e.g., heart rate at peak stress, exercise metabolic equivalents, and exercise time [19]. The performance of their model was compared with other scores, such as CHARGE-AF, FHS and ARIC [14,20,21], and it was significantly higher, taking into account specificity, sensitivity, and area under the ROC curve. Comparison with other scales in the Polish population can be a valuable approach, thus we plan to improve our research in this field. 

## 5. Limitations

Even though the results of the presented research are clinically relevant, the study has several limitations. Its retrospective design limits the availability of data on clinical characteristics. The study was performed without a control group; due to the rarity of the disease, we aimed to extend the number of participants in the study group. Additionally, the time when a diagnosis of HCM was made is distant, but the appropriate follow-up period was necessary to ensure the relevance of the data. However, it is crucial to mention that there was no loss to the follow-up after the first evaluation at the Clinic. Furthermore, we cannot ensure that every episode of atrial fibrillation occurring during the follow-up period was recorded, due to possibly insufficient AF screening that should be performed mainly at the general practitioner’s office, not at the tertiary care facility. Although the mean age of the studied group, both at evaluation and at the diagnosis, is similar to the original population analysed by Carrick et al. (age at evaluation 53.5 ± 13.6, vs. 49 ± 16 years; age at diagnosis 46.5 ± 15.9, vs. 45 ± 17) it is necessary to assess the risk of AF occurrence at an earlier age, because of its significant burden on HCM patients’ quality of life and life expectancy. Nonetheless, to address the matter of advanced age at the time of the HCM diagnosis, it is vital to improve diagnostic tools and utilise genetic testing for hereditary cardiovascular diseases at large. It should be also noted that the analysed group was not assessed using the Sudden Cardiac Death Risk score (HCM-Risk SCD Score) [13]. This scale was not used, due to the insufficient data available. Nevertheless, the study endpoints taken into account were of a different character, corresponding to AF and its consequences.

## 6. Conclusions

Regardless of the fact that Polish patients with HCM are characterised by a relatively high HCM-Risk Score, the score underestimates the real-life high level of AF incidence. The HCM-AF Risk Score seems to be useful in the prediction of general clinical outcomes in HCM patients. It allows the distinction of HCM patients with high mortality and rehospitalisation due to HF.

## Figures and Tables

**Table 1:** Demographic and clinical characteristics of patients at baseline.

	Overall N = 92 (100%)	Low Risk N = 13 (14.1%)	Intermediate Risk N = 30 (32.6%)	High Risk N = 49 (53.3%)
sex—male N (%)	46 (50.0)	7 (53.8)	14 (46.7)	25 (51.0)
age at HCM diagnosis [years]	46.5 ± 15.9	31.7 ± 13.1 *	44.2 ± 11.1	51.8 ± 15.4
NYHA class				
class I N (%)	29 (31.5%)	12 (92.3%) *	12 (40.0%)	5 (10.2%)
class II N (%)	37 (40.2%)	1 (7.7%) *	11 (36.7%)	25 (51.0%)
class III N (%)	21 (22.8%)	0 (0%) *	6 (20.0%)	15 (30.6%)
class IV N (%)	5 (5.5%)	0 (0%) *	1 (3.3%)	4 (8.2%)
BMI [kg/m^2^]	26.1 ± 5.3	24.9 ± 3.8 *	24.5 ± 5.5	29.1 ± 4.6
TTE baseline parametes:				
LV EDD [mm]	48.1 ± 7.8	45.8 ± 5.3 *	46.2 ± 8.2	49.0 ± 7.5
IVSd [mm]	17.5 ± 5.2	18.5 ± 7.7	17.9 ± 5.0	17.9 ± 4.0
PWd [mm]	11.3 ± 2.7	10.3 ± 2.7	11.6 ± 2.6	11.8 ± 2.7
LVMI [g/m^2^]	160.0 ± 54.1	153.8 ± 70.4	144.7 ± 53.4	172.8 ± 48.1
LV EF (%)	52.3 ± 13.1	56.2 ± 3.5	57.7 ± 9.1	50.6 ± 13.6
LA diameter [mm]	43.8 ± 8.8	36 ± 6.4 *	39.7 ± 4.3	49.9 ± 9.6
LA area [cm^2^]	26.9 ± 8.2	20.5 ± 4.2 *	27.1 ± 8.0	32.3 ± 8.1
nsVT N (%)	22 (23.9%)	5 (38.5%) *	5 (16.7%)	6 (12.4%)
ICD implantation during follow-up	30 (31.6%)	4 (75.0%) *	5 (16.7%)	21 (42.9%)

BMI, body-mass index; ICD, implantable cardioverter defibrillator; IVSd, intraventricular septum diameter; LA, left atrium; LVEDD, left ventricle end diastolic diameter; LVEF, left ventricle ejection fraction; LVMI, left ventricular mass index; NSVT, non-sustained ventricular tachycardia; NYHA, New York Heart Association; PWd, posterior wall diameter; * denotes statistically significant difference (*p* < 0.05) among the subgroups.

**Table 2:** Comparison of risk for AF predicted using the HCM-AF Risk Score vs. the registered AF incidence, in all risk subgroups.

Subgroup		HCM-AF Estimated Risk	Registered AF Incidence
Low risk (N = 13)	2-year follow-up	1.1%	7.7%
	5-year follow-up	2.6%	15.4%
Intermediate Risk (N = 30)	2-year follow-up	3.5%	16.7%
	5-year follow-up	8.3%	40.0%
High risk (N = 49)	2-year follow-up	13.6%	46.9%
	5-year follow-up	29.0%	51.0%

**Table 3:** Primary and secondary endpoints registered for the overall population and all risk subgroups.

		Overall (N = 92)	Low Risk (N = 13)	Intermediate Risk (N = 30)	High Risk (N = 49)
Primary endpoint	AF at 2-year	29 (31.5%)	1 (7.7%) *	5 (16.7%)	23 (46.9%)
	AF at 5-year	40 (43.5%)	2 (15.4%) *	12 (40.0%)	25 (51.0%)
Secondary endpoints	total mortality	23 (42.6%)	2 (15.4%) *	6 (20.0%)	21 (42.9%)
	rehospitalisation due to AF	54 (58.7%)	3 (23.1%) *	16 (53.3%)	35 (71.4%)
	complex: total mortality and rehospitalisation due to HF	59 (64.1%)	3 (23.1%) *	18 (60.0%)	38 (77.6%)
Other data on clinical outcomes	HF progression	25 (27.2%)	0 (0.0%) *	7 (23.3%)	18 (36.7%)
	no change in HF	51 (55.4%)	13 (100.0%) *	17 (56.7%)	21 (42.9%)
	HF regression	16 (17.4%)	0 (0.0%)	6 (20.0%)	10 (20.4%)

AF, atrial fibrillation; HF, heart failure; * denotes statistically significant difference (*p* < 0.05) among the subgroups.

## Data Availability

The source data of the article is available upon request.
